# A novel prognostic signature based on cancer stemness and metabolism-related genes for cervical squamous cell carcinoma and endocervical adenocarcinoma

**DOI:** 10.18632/aging.205757

**Published:** 2024-04-23

**Authors:** Yaokai Wang, Yuanyuan Han, Liangzi Jin, Lulu Ji, Yanxiang Liu, Min Lin, Sitong Zhou, Ronghua Yang

**Affiliations:** 1 Department of Gynecology and Obstetrics, The University of Hong Kong-Shenzhen Hospital, Shenzhen 518053, Guangdong, China; 2 Institute of Medical Biology, Chinese Academy of Medical Sciences and Peking Union Medical College, Yunnan Key Laboratory of Vaccine Research and Development on Severe Infectious Diseases, Kunming, Yunnan, China; 3 Yantian District Maternal and Child Health Hospital, Shenzhen, Guangdong, China; 4 Department of Dermatology, The First People’s Hospital of Foshan, Foshan, Guangdong, China; 5 Department of Burn and Plastic Surgery, Guangzhou First People’s Hospital, South China University of Technology, Guangzhou, Guangdong, China

**Keywords:** cervical carcinoma, mRNAsi, metabolism-related gene signatures, WGCNA, bioinformatic analysis

## Abstract

Background: CESC is the second most commonly diagnosed gynecological malignancy. Given the pivotal involvement of metabolism-related genes (MRGs) in the etiology of multiple tumors, our investigation aims to devise a prognostic risk signature rooted in cancer stemness and metabolism.

Methods: The stemness index based on mRNA expression (mRNAsi) of samples from the TCGA dataset was computed using the One-class logistic regression (OCLR) algorithm. Furthermore, potential metabolism-related genes related to mRNAsi were identified through weighted gene co-expression network analysis (WGCNA). We construct a stemness-related metabolic gene signature through shrinkage estimation and univariate analysis, thereby calculating the corresponding risk scores. Moreover, we selected corresponding DEGs between groups with high- and low-risk score and conducted routine bioinformatic analyses. Furthermore, we validated the expression of four hub genes at the protein level through immunohistochemistry (IHC) in samples obtained from our patient cohort.

Results: According to the findings, it was found that six genes—AKR1B10, GNA15, ALDH1B1, PLOD2, LPCAT1, and GPX8— were differentially expressed in both TCGA-CSEC and GEO datasets among 23 differentially expressed metabolism-related genes (DEMRGs). mRNAsi exhibited a notable association with the extent of key oncogene mutation. The results showed that the AUC values for forecasting survival at 1, 3, and 5 years are 0.715, 0.689, and 0.748, individually. We observed a notable association between the risk score and different immune cell populations, along with enrichment in crucial signaling pathways in CESC. Four genes differentially expressed between different risk score groups were validated by IHC to be highly expressed in the CESC samples at the protein level.

Conclusion: The current investigation indicated that a 3-gene signature based on stemness-related metabolic and 4 hub genes with differential expression between high and low-risk score subgroups may serve as valuable prognostic markers and potential therapeutic targets in CESC.

## INTRODUCTION

According to the 2020 Global Cancer Statistics, CESC is the second most commonly diagnosed gynecological malignancy and the leading cause of death among women [1]. However, many developing countries lack vaccination and screening programs, which increases the difficulty of diagnosis and treatment [2, 3]. Each year, approximately 604,000 new cases of CESC are diagnosed, with 342,000 resulting in mortality [1]. In most cases, patients are often diagnosed at the moderate or advanced stages of the disease. While current treatment strategies, such as surgery, radiotherapy, and chemotherapy, are promising for CESC patients, about 75% of patients would experience disease progression and/or recurrence [4]. Therefore, there is an imperative need to investigate the carcinogenic mechanisms underlying CESC and develop a novel prognostic model for this malignancy.

Cancer stem cells (CSCs) have become attractive targets for cancer treatment due to their capacity for self-renewal and multi-lineage differentiation, which contribute to tumor growth and heterogeneity. CSCs are more aggressive than normal cancer cells, thereby promoting tumor invasion and metastasis [5]. In recent years, a new cancer stemness index (mRNAsi) generated by deep learning method has gained significant attention in diverse cancers [6], including hepatocellular carcinoma [7, 8], renal cell carcinoma [8], lung cancer [9], and glioma [10]. Furthermore, metabolic reprogramming has emerged as a novel fundamental characteristic of cancer cells in recent years [11]. Increased glycolysis under normoxic conditions (known as the Warburg effect) and alterations in glutamine metabolism represent prominent metabolic adaptations in tumor cells. Emerging data suggest that aberrant metabolism is correlated with unfavorable clinical outcomes across various tumor types, including cervical carcinoma [12]. Nevertheless, the precise involvement and mechanisms underlying the interplay between stemness and metabolism in the progression and prognosis of CESC remain inadequately elucidated. Furthermore, there is a lack of prognostic models utilizing stemness- and metabolism-related genes to predict outcomes in CESC patients.

**Figure 1:**
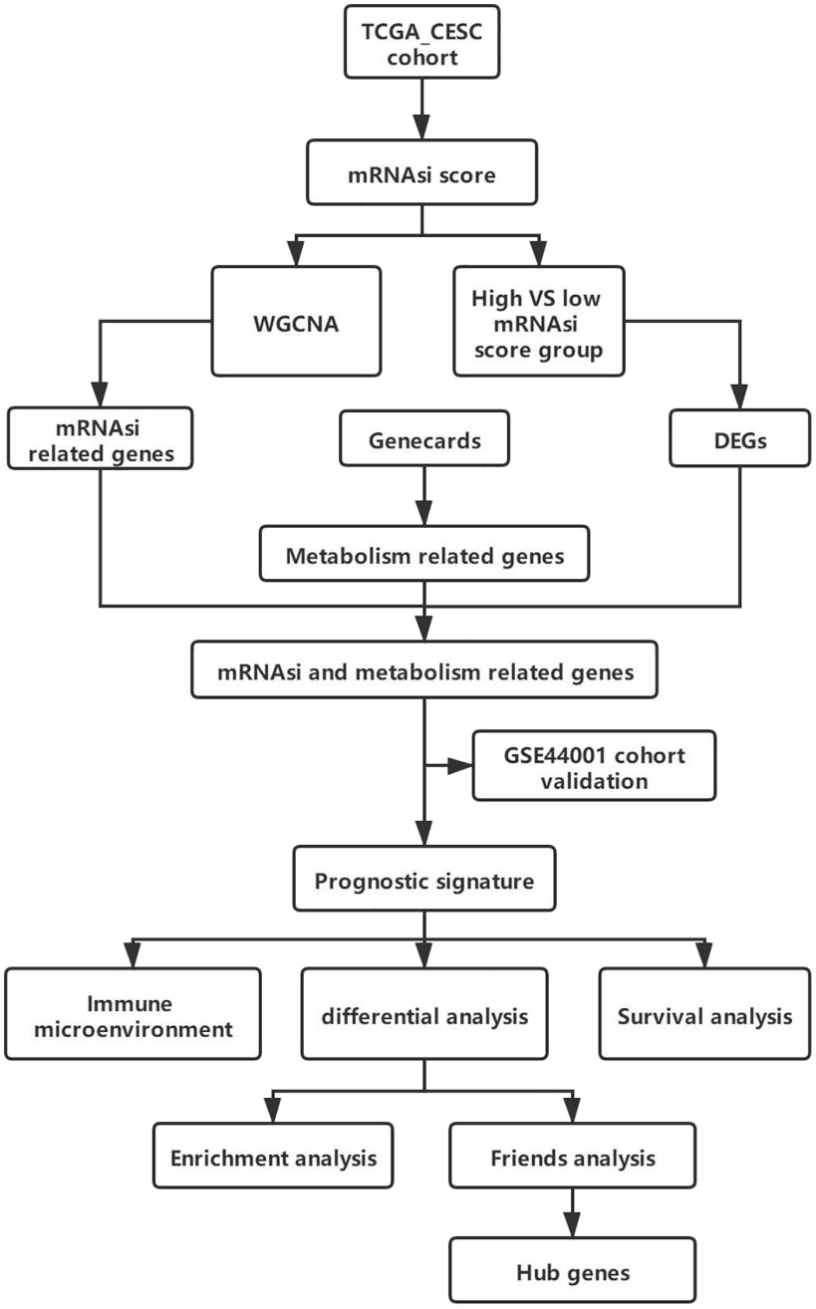
Overall experimental flow chart.

This study intended to establish a prognostic model of differentially expressed metabolism-associated genes (DEGs) linked to stem cell properties, utilizing mRNAsi as a basis in CESC. All samples were categorized into high- and low-mRNAsi score subgroups based on mRNAsi score. Then we utilized WGCNA [13] to explore cancer stemness characteristics and find out mRNAsi score-related DEGs. We constructed a new prognostic gene signature for CESC, integrating cancer stemness and metabolism, through comprehensive univariate and multivariate Cox regression analyses. Subsequently, we validated the expression of these signature genes using external GEO datasets. Additionally, we extensively explored the interplay among cancer stemness, immune microenvironment, and gene expression differences while conducting survival analyses stratified by risk scores. Our findings shed light on the associations between mRNAsi, immune cell infiltration, and prognostic implications. We further identified eight hub DEGs distinguishing high- from low-risk score groups. Among these genes, four hub genes were further validated through IHC analysis using patient samples. A schematic overview of the experimental workflow was depicted (Figure 1), illustrating the feasibility of our approach for application in other cancer research endeavours. Our work could provide insights for the potential mechanism of CESC and stemness-related metabolic targets while also offering targets for precise immunotherapy targeting stemness-related metabolic pathways.

## RESULTS

### Data pre-processing and screening of metabolic genes related to mRNAsi- (MRGS)

**Figure 2:**
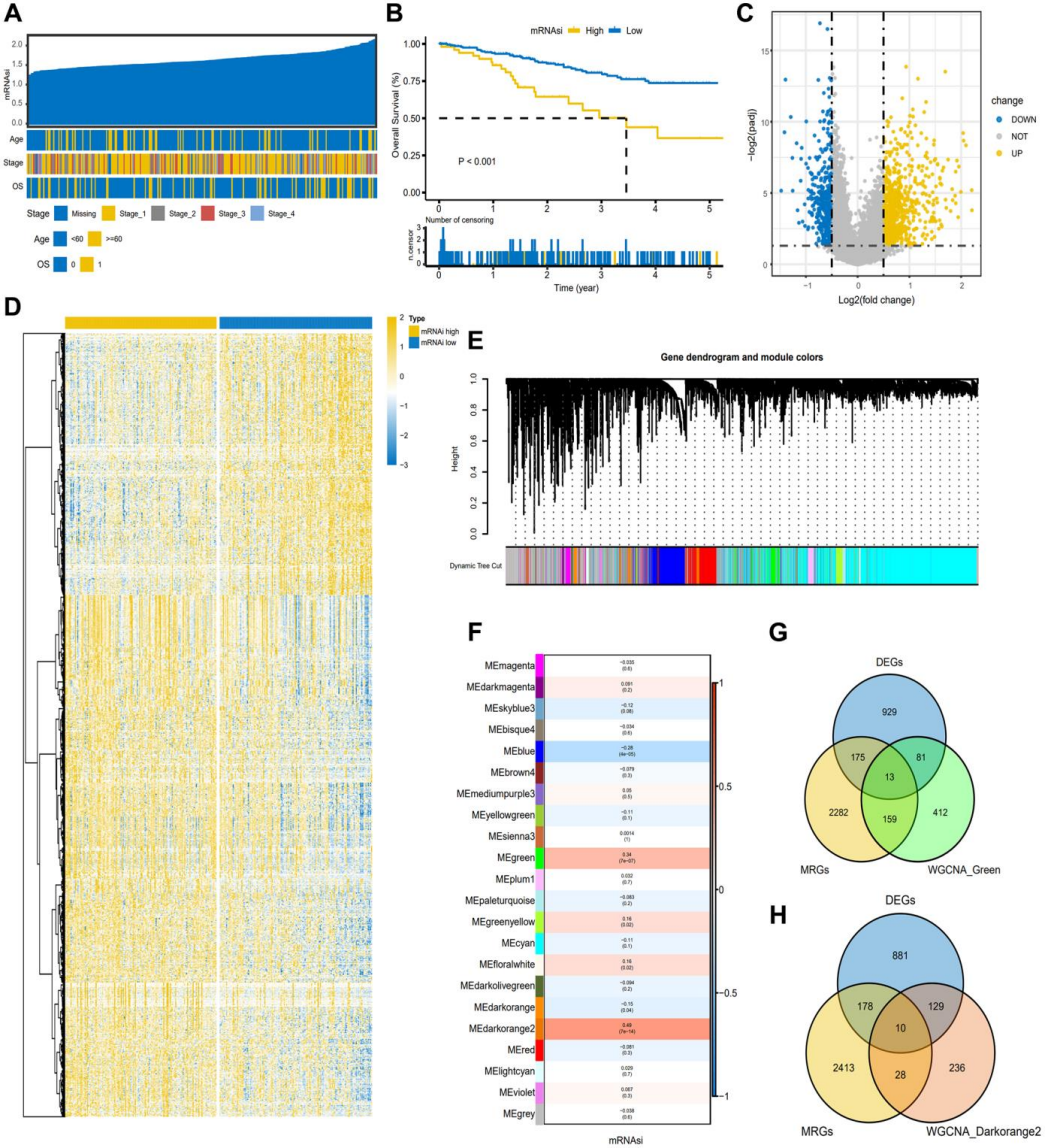
**Screening of candidate genes based on TCGA-CESC.** (**A**) Overview of the association between known clinical and molecular features and mRNAsi in CESC. The list shows the samples sorted by mRNAsi from low to high. Rows represent known clinical and molecular characteristics. (**B**) The K-M plot showed the OS of CESC patients with high or low mRNAsi. (**C**) The heat map of differentially expressed genes grouped according to the level of mRNAsi. (**D**) The volcano map of differentially expressed genes grouped according to the level of mRNAsi. (**E**) Different modules obtained by WGCNA clustering. (**F**) Cluster Heatmap showed the correlation and significant difference between gene module and mRNAsi score. The *p*-value is shown in parentheses. (**G**, **H**). Venn diagram of the intersection of genes in green and darkorange2 modules with metabolism-related genes (MRGS) and differentially expressed genes, respectively.

Firstly, the gene expression matrix of TCGA-CESC was transformed into TPM data, and the mRNAsi value of each sample was obtained using the GSVA method. CESC samples were sorted based on their mRNAsi scores, ranging from low to high stemness index, and subsequently examined for potential association with demographic, molecular, or clinical characteristics (Figure 2A). Survival analysis identified a notable impact of mRNAsi on the overall survival (OS) of CESC (*P* < 0.001) (Figure 2B). According to the level of mRNAsi, DEGs were selected from TCGA-CESC gene dataset through Deseq2 package in the R software, as shown in the volcano map and heatmap (Figure 2C, 2D). At the same time, we built a WGCNA co-expression network in order to determine the gene modules with biological significance and further identify the genes proximately related to the stemness of CESC cells (mRNAsi). A total of 22 modules were acquired for the following analysis. Module significance (MS) was computed to determine the relationship between mRNAsi score and genes (Figure 2E). Since an R2 value close to 1 indicated a strong connection between GC dryness and gene expression. As shown in Figure 2F, we screened two modules exhibiting the most robust correlation and considered that they have a strong correlation with CESC dryness, namely the green module and the darkorange2 module. The green module (R2 = 0.34, *P* < 0.001) and darkorange2 module was positively correlated with mRNAsi (R2 = 0.49, *P* < 0.001). The genes in the two modules were overlapped with the previously identified DEGs and known metabolism-related genes (MRGS), visualized using Wayne diagram (Figure 2G, 2H). 13 and 10 candidate genes were obtained for subsequent analysis, respectively.

### Validation of mRNAsi-related metabolic genes in the GEO dataset

**Figure 3:**
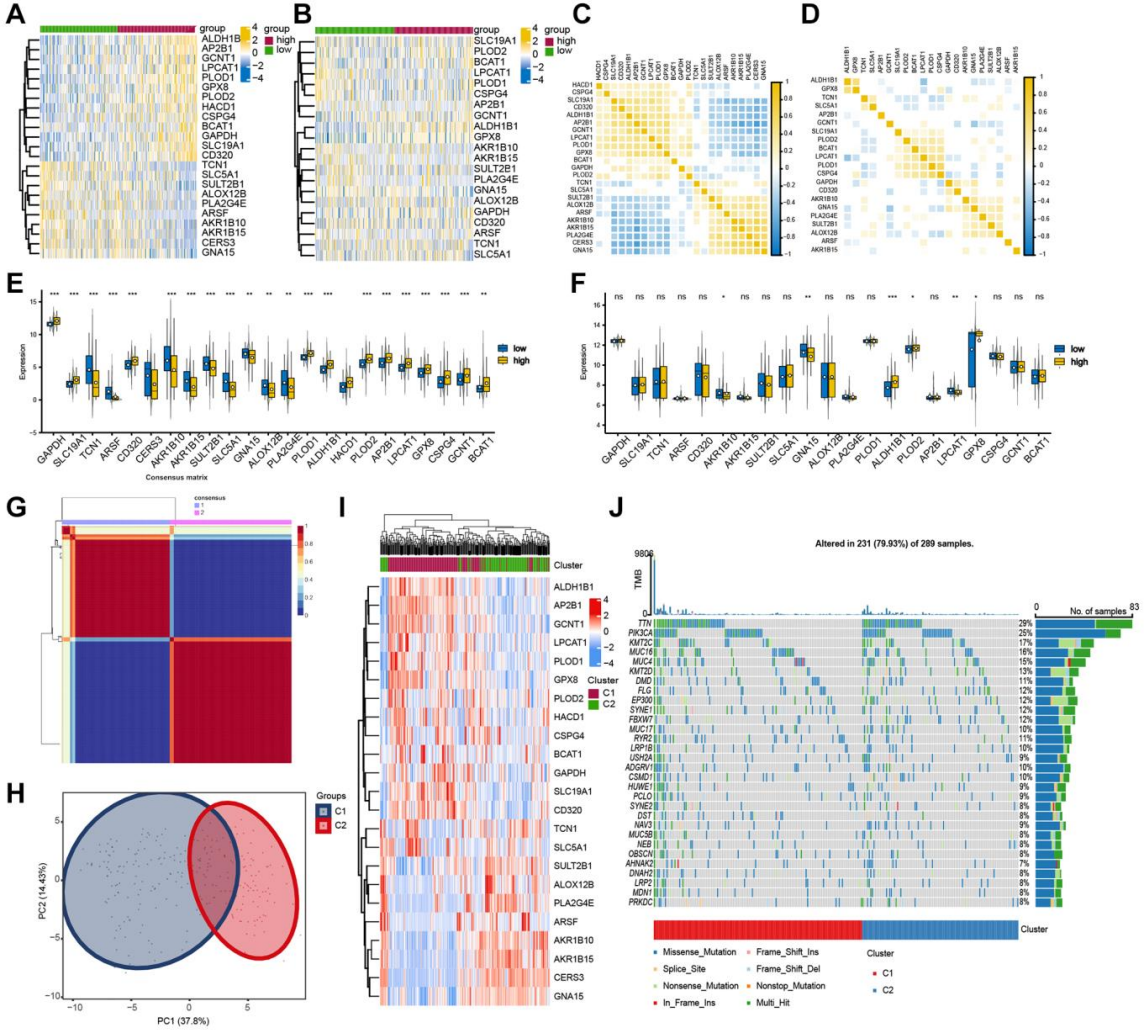
**Validation of screened gene set in TCGA-CESC dataset.** (**A**, **B**) Differential heat map of candidate gene set in TCGA-CESC dataset and GSE44001. (**C**, **D**) The correlation heat map of candidate genes in TCGA-CESC and GSE44001. The non-statistically significant mutual modules are displayed in blank. (**E**, **F**) Differential expression of candidate genes in TCGA-CESC and GSE44001. (**G**) NMF consistent clustering of screened gene sets in TCGA-CESC. (**H**) PCA analysis of two subgroups. (**I**) Heat map of candidate gene expression between the two subgroups. (**J**) The mutation landscape waterfall plot between two subgroups of TCGA-CESC.

We extracted the genes expression matrix from the two modules by R software, and the differential expression profile of these 23 candidate genes between high- and low-mRNAsi groups from both TCGA-CESC and GSE44001 datasets were illustrated in a heatmap (Figure 3A, 3B). The correlation heatmap was used to show the correlation among these 23 candidate genes expression (Figure 3C, 3D). Then, the differential expression of the 23 candidate genes in the two datasets were further shown by grouped box chart, and it was found that AKR1B10, GNA15, ALDH1B1, PLOD2, LPCAT1, and GPX8 genes exhibited differential expression in the two datasets. (Figure 3E, 3F).

Then, the gene expression matrix of TCGA-CESC was clustered by NMF consistent clustering (Figure 3G). According to the mean expression level of the chosen candidate genes in each sample, the samples were divided into two subgroups (cluster1 and cluster2). The sample differentiation of the two subgroups were displayed by PCA analysis. The distribution of 23 candidate genes in the two subgroups were displayed by heatmap (Figure 3H, 3I). Finally, to investigate the mutation characteristics of distinct subgroups, we examined the mutant genes between the two subgroups as illustrated by the mutation waterfall diagram. We found that the significant mutant genes between the two subgroups were TTN, PIK3CA, KMT2C, MUC16 (Figure 3J).

### Development of a prognostic model utilizing the candidate genes

**Figure 4:**
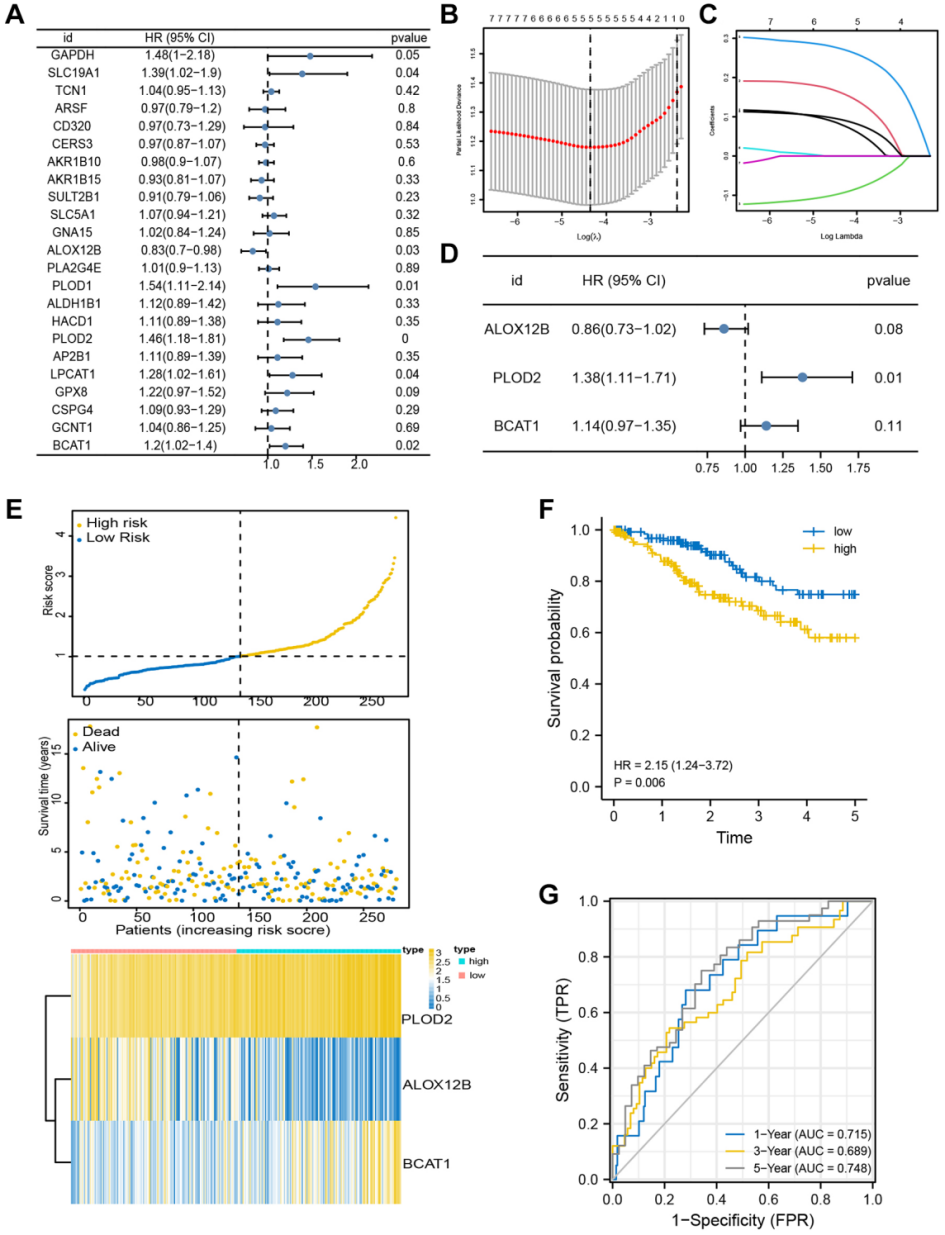
**Identification of prognosis-related genes.** (**A**) Univariate Cox analysis was used to double-screen the prognosis-related genes in the screened gene set. (**B**) Parameter selection in lasso model is 100 times cross validation. (**C**) Lasso coefficient spectrum of prognostic gene screening. (**D**) Stepwise Cox proportional hazards regression model was used to further screen the prognosis-related genes. (**E**) Risk score distribution, survival status, and gene expression profile. (**F**, **G**) K-M survival plot and ROC analysis for predicting 1-year, 3-year and 5-year prognosis.

Here in the TCGA-CESC assembly, a univariate Cox proportional regression model screened out eight genes associated with OS (*p* < 0.1) (Figure 4A). Next, the Lasso Cox regression model was used to screen prognostic markers. A standard error (SE) higher than the minimum standard was selected to obtain a model containing five genes (Figure 4B, 4C). In order to optimize the model and include the most prognostically relevant genes, we employed a stepwise Cox proportional hazards regression model. This approach led to the identification of the final three genes, with special emphasis on PLOD2 as an independent prognostic gene (Figure 4D). The distribution of risk score, survival status, and gene expression profiles were illustrated (Figure 4E). Then, the patients were stratified into the high-score group and low-score group based on the optimized risk score. Kaplan Meier survival analysis revealed a notable higher survival rate in the high-score group compared to the low-score group (Figure 4F, *P* = 0.006). Our findings demonstrated AUC values of 0.715, 0.689, and 0.748 for predicting survival at 1, 3, and 5 years, respectively (Figure 4G), suggesting a robust predictive efficacy of this model.

### DEGs selection and functional enrichment analysis utilizing risk score grouping

**Figure 5:**
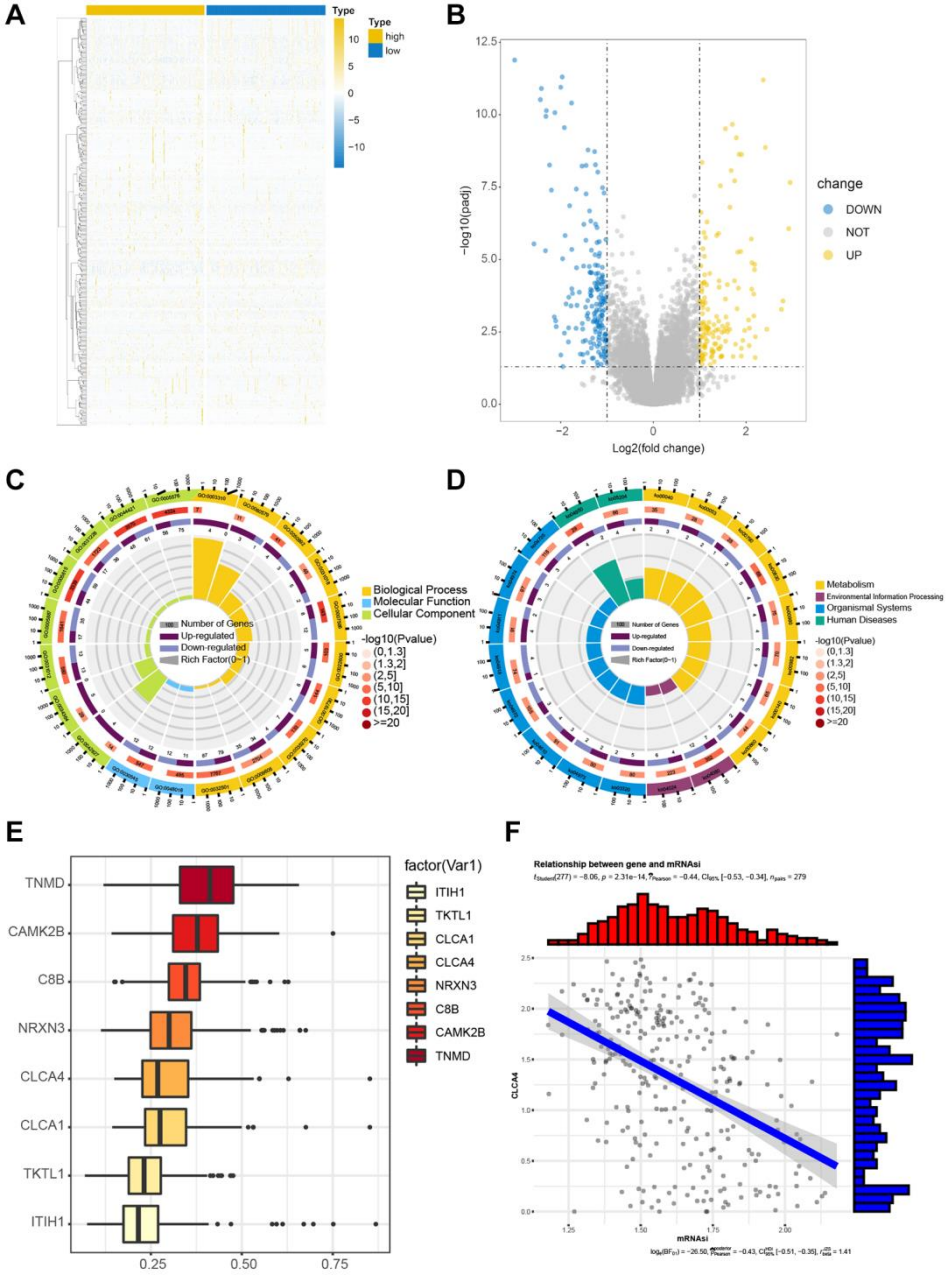
**Functional enrichment of DEGs in TCGA-CESC cohort.** The correlation between the expression levels of candidate genes. (**A**, **B**) The heat map and volcano map for screened DEGs by risk score grouping. (**C**) Significant enrichment results of GO function. (**D**) Significantly enriched KEGG pathway. (**E**) Semantic similarity of GO terms of eight core genes by FRIENDS analysis. (**F**) The correlation between CLCA4 and mRNAsi was statistically significant.

We divided the samples according to the above risk score, then extracted corresponding DEGs between the high- and low-risk score groups, as illustrated using the volcano map and heatmap (Figure 5A, 5B). Then, the DEGs were analyzed by pathway enrichment and GO enrichment, respectively. Enriched cell component (CC) included the extracellular region, extracellular space, integral component of plasma membrane, and chylomicron (Figure 5C). The pathway enrichment analysis suggested notable enrichment of genes in pathways related to retinol metabolism, neuroactive live receptor interaction, maturity onset diabetes of the young, metabolism of xenobiology by cytochrome P450, and chemical carcinogenesis pathways (Figure 5D). Eight hub genes were screened by semantic similarity analysis between GO terms (Friends analysis) (Figure 5E). Especially, it was found that CLCA4 was negatively associated with mRNAsi score (*P* < 0.01, r = −0.44) (Figure 5F). The detailed GO and KEGG results were shown in Tables 1 and 2.

**Table 1:** GO analysis of DEGs.

**GO ID**	**Description**	***p*.adjust**
GO:0005576	Extracellular region	5.50E-13
GO:0044421	Extracellular region part	2.15E-10
GO:0005615	Extracellular space	4.71E-10
GO:0031012	Extracellular matrix	1.20E-04
GO:0005887	Integral component of plasma membrane	1.20E-04
GO:0031226	Intrinsic component of plasma membrane	1.89E-04
GO:0042627	Chylomicron	3.62E-03
GO:0034364	High-density lipoprotein particle	4.91E-03
GO:0044459	Plasma membrane part	9.52E-03
GO:0034366	Spherical high-density lipoprotein particle	9.58E-03
GO:0034361	Very-low-density lipoprotein particle	1.05E-02
GO:0034385	Triglyceride-rich plasma lipoprotein particle	1.05E-02
GO:0034358	Plasma lipoprotein particle	1.05E-02
GO:1990777	Lipoprotein particle	1.05E-02
GO:0005903	Brush border	1.09E-02
GO:0032994	Protein-lipid complex	1.32E-02
GO:0008076	Voltage-gated potassium channel complex	1.58E-02
GO:0098855	HCN channel complex	1.82E-02
GO:0099699	Integral component of synaptic membrane	1.82E-02
GO:0034705	Potassium channel complex	2.37E-02
GO:0005796	Golgi lumen	2.54E-02
GO:0098862	Cluster of actin-based cell projections	2.69E-02
GO:0099240	Intrinsic component of synaptic membrane	2.80E-02
GO:0072562	Blood microparticle	2.92E-02
GO:0045177	Apical part of cell	2.94E-02
GO:0016324	Apical plasma membrane	3.94E-02
GO:0044306	Neuron projection terminus	4.06E-02
GO:0030141	Secretory granule	4.48E-02
GO:0099055	Integral component of postsynaptic membrane	4.48E-02
GO:0031091	Platelet alpha granule	4.81E-02

**Table 2:** KEGG analysis of DEGs.

**ID**	**ID**	**Class**	***P*-value**
ko00830	Retinol metabolism	Metabolism	1.90E-06
ko04950	Maturity onset diabetes of the young	Human Diseases	7.38E-06
ko00980	Metabolism of xenobiotics by cytochrome P450	Metabolism	3.64E-05
ko05204	Chemical carcinogenesis	Human Diseases	9.78E-05
ko00982	Drug metabolism - cytochrome P450	Metabolism	1.71E-04
ko00040	Pentose and glucuronate interconversions	Metabolism	2.82E-04
ko04972	Pancreatic secretion	Organismal Systems	3.92E-04
ko03320	PPAR signaling pathway	Organismal Systems	3.94E-04
ko00140	Steroid hormone biosynthesis	Metabolism	7.78E-04
ko04610	Complement and coagulation cascades	Organismal Systems	8.60E-04
ko00053	Ascorbate and aldarate metabolism	Metabolism	1.18E-03
ko00790	Folate biosynthesis	Metabolism	1.18E-03
ko04725	Cholinergic synapse	Organismal Systems	3.84E-03
ko04911	Insulin secretion	Organismal Systems	4.38E-03
ko04974	Protein digestion and absorption	Organismal Systems	5.98E-03
ko00860	Porphyrin and chlorophyll metabolism	Metabolism	6.37E-03
ko04918	Thyroid hormone synthesis	Organismal Systems	8.26E-03
ko04973	Carbohydrate digestion and absorption	Organismal Systems	9.99E-03
ko00983	Drug metabolism - other enzymes	Metabolism	1.14E-02
ko04935	Growth hormone synthesis, secretion and action	Organismal Systems	1.87E-02
ko04970	Salivary secretion	Organismal Systems	2.07E-02
ko04923	Regulation of lipolysis in adipocyte	Organismal Systems	2.18E-02
ko04929	GnRH secretion	Organismal Systems	2.67E-02
ko00350	Tyrosine metabolism	Metabolism	2.80E-02
ko00730	Thiamine metabolism	Metabolism	2.93E-02
ko04713	Circadian entrainment	Organismal Systems	2.94E-02
ko04915	Estrogen signaling pathway	Organismal Systems	3.26E-02
ko04924	Renin secretion	Organismal Systems	3.51E-02
ko00360	Phenylalanine metabolism	Metabolism	3.66E-02
ko04922	Glucagon signaling pathway	Organismal Systems	3.76E-02
ko05218	Melanoma	Human Diseases	3.98E-02
ko04971	Gastric acid secretion	Organismal Systems	4.15E-02

### GSEA analysis of metabolic-related pathways related to risk scores

**Figure 6:**
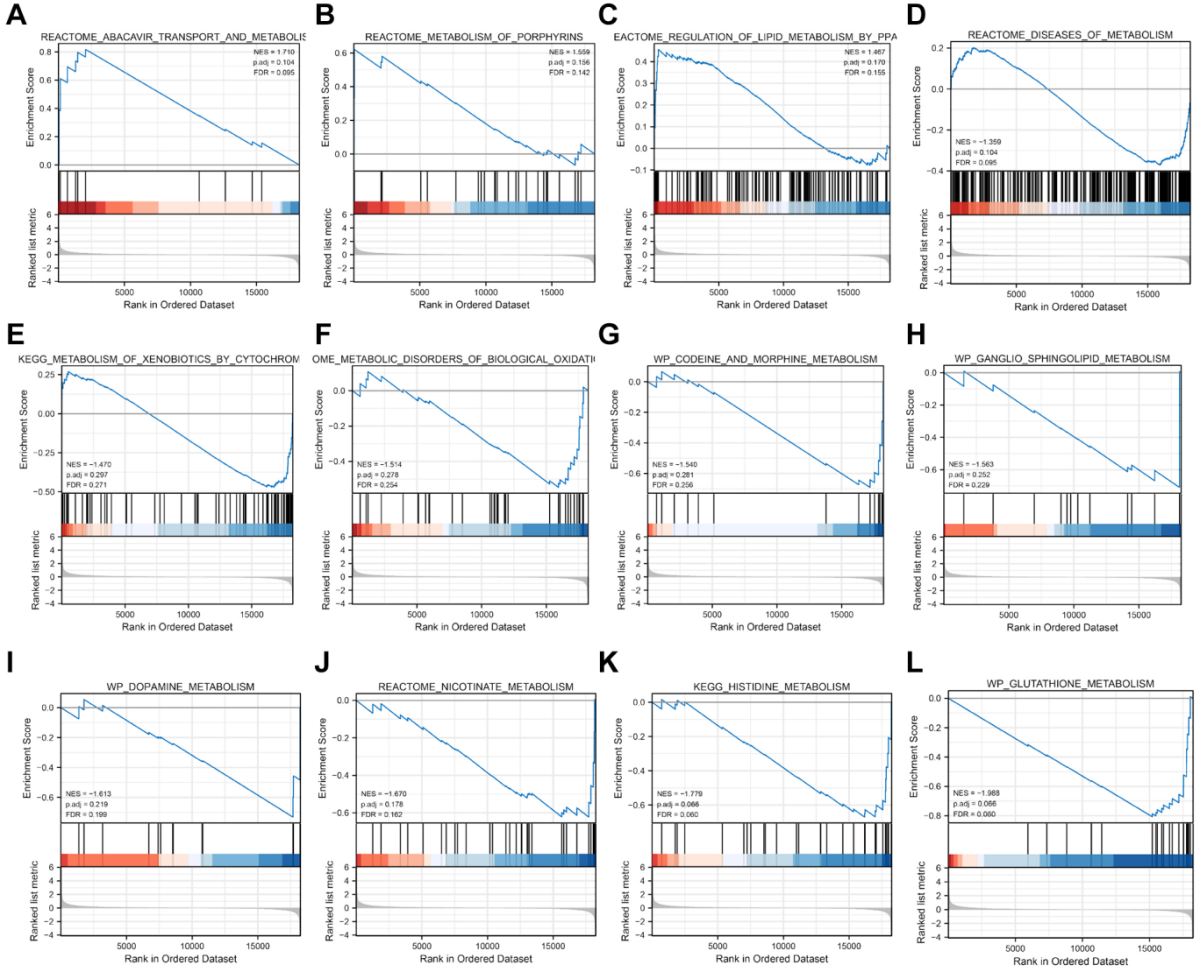
**Metabolic pathways of GSEA enrichment analysis related to risk score.** (**A**–**H**) The detailed information of GSEA metabolic pathways.

GSEA was conducted to compare pathway enrichment between the high-risk and low-risk groups in the TCGA-CESC dataset, utilizing the risk score to identify pathways of significant enrichment (*P*-value < 0.05). Additionally, 12 significant enrichment pathways were found to be related to metabolism, including reactome_ ABACAVIR_TRANSPORT_AND_METABOLISM; REACTOME_METABOLISM_OF_PORPHYRINS, REACTOME_REGULATION_OF_LIPID_METABOLISM_BY_PPARalpha showed significant enrichment in the high-risk score group. REACTOME_DISEASES_OF_METABOLISM, KEGG_METABOLISM_OF_XENOBIOTICS_BY_CYTOCHROME_P450; REACTOME_METABOLIC_DISORDERS_OF_BIOLOGICAL_OXIDATION_ENZYMES; WP_CODEINE_AND_MORPHINE_METABOLISM; WP_GANGLIO_SPHINGOLIPID_Metabolism and others showed significant enrichment in the low-risk score group. (Figure 6A–6H). The detailed GSEA results were shown in Table 3.

**Table 3:** GSEA results.

**ID**	**NES**	***P*-value**	**Rank**
REACTOME_ABACAVIR_TRANSPORT_AND_METABOLISM	1.71	5.54E-03	2051
REACTOME_METABOLISM_OF_PORPHYRINS	1.56	1.07E-02	32
REACTOME_REGULATION_OF_LIPID_METABOLISM_BY_PPARALPHA	1.47	1.23E-02	322
REACTOME_DISEASES_OF_METABOLISM	−1.36	5.17E-03	2298
KEGG_METABOLISM_OF_XENOBIOTICS_BY_CYTOCHROME_P450	−1.47	3.59E-02	1465
REACTOME_METABOLIC_DISORDERS_OF_BIOLOGICAL_OXIDATION_ENZYMES	−1.51	3.15E-02	2294
WP_CODEINE_AND_MORPHINE_METABOLISM	−1.54	3.25E-02	1048
WP_GANGLIO_SPHINGOLIPID_METABOLISM	−1.56	2.56E-02	121
WP_DOPAMINE_METABOLISM	−1.61	1.92E-02	563
REACTOME_NICOTINATE_METABOLISM	−1.67	1.36E-02	533
KEGG_HISTIDINE_METABOLISM	−1.78	2.26E-03	2054
WP_GLUTATHIONE_METABOLISM	−1.99	2.24E-03	3031

### Variations in immune microenvironment between the high- and low-risk score groups

**Figure 7:**
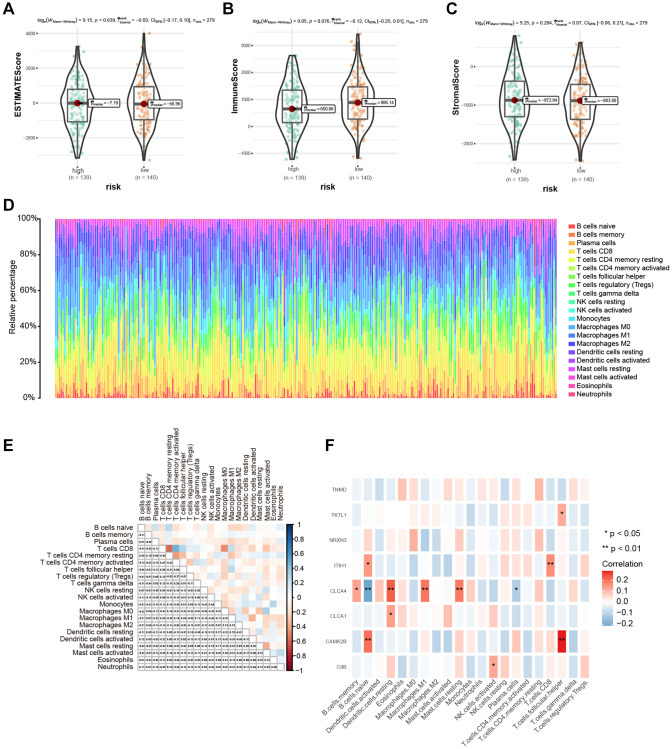
**Immune microenvironment assessment based on risk score.** (**A**–**C**) The difference in immune microenvironment score between different risk groups. (**D**) The histogram sorted according to the risk score showed the distribution of 22 immune infiltrating cells. (**E**) Correlation analysis of 22 kinds of immune cells. (**F**) Heat map of correlation between hub genes and 22 kinds of immune cell infiltrations; Red indicated positive correlation, blue indicated negative correlation, and the darker the color, the stronger the correlation. ^*^P < 0.05, ^**^P < 0.01, ^***^P < 0.001.

The estimate method was used to determine tumor purity, matrix and immune score to study the correlation between high- and low-risk groups. The two risk groups did not differ significantly (Figure 7A–7C). Then, we ranked the samples according to the risk score and displayed the infiltration of immune cells in each sample from the TCGA cohort using a histogram. The scores representing the infiltration of 22 immune cells computed using CIBERSORT algorithm are shown in Figure 7D, and the associations among immune cells are shown in Figure 7E. Additionally, after calculating the association between the eight hub genes and immune cell infiltration, we found out a notable correlation between the CLCA4 gene and a variety of immune cells (B memory cells, B naïve cells, resting dendritic cells, M1 macrophages, resting mast cells, and plasma cells), which may suggest that CLCA4 gene exhibits significant involvement in tumor immunity within CESC (Figure 7F).

### Histologic examination

**Figure 8:**
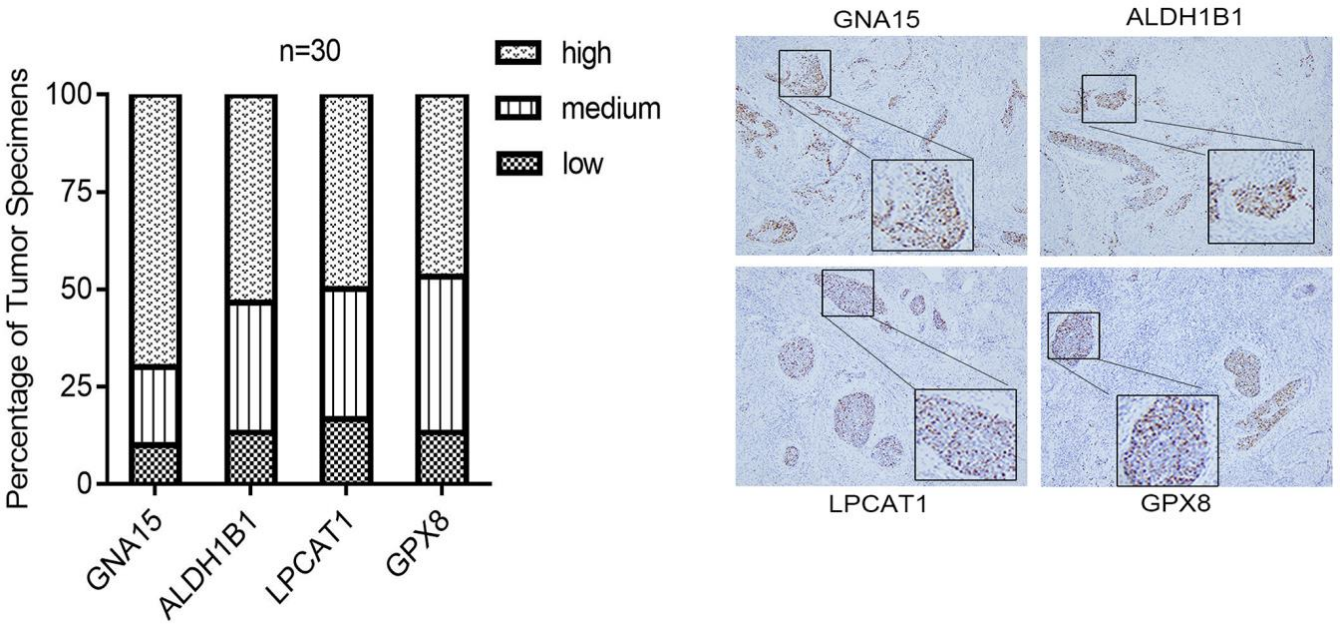
The IHC staining validation results of the hub genes, including GNA15, ALDH1B1, LPCAT1, and GPX8, in CESC tissues.

We next investigated the protein expression level of the hub genes, including GNA15, ALDH1B1, LPCAT1, and GPX8, in CESC tissues. The IHC staining results revealed that four genes displayed high-level expressions in 30 CESC specimens (Figure 8). In CESC patients, the rates of high expression of GNA15, ALDH1B1, LPCAT1, and GPX8 were 70%, 53.3%, 50.0%, and 46.7%, respectively. The rates of low expression of GNA15, ALDH1B1, LPCAT1, and GPX8 were 10.0%, 13.3%, 16.7%, and 13.3%, respectively.

## DISCUSSION

Recent studies have indicated a positive association between the presence of cancer stem cells (CSCs) and regulatory T cells (Treg) in cancer, suggesting that targeting the interaction between Treg and CSCs holds promise as a therapeutic strategy [14]. Furthermore, metabolic abnormalities have been linked to unfavourable outcomes in various types of tumors, such as Hela. Hence, gaining deeper insights into personalized stemness and metabolism-related signatures alongside tumor immunology of CSCs could potentially offer highly effective therapeutic avenues within the framework of immunotherapy strategies [15].

In the current study, mRNAsi was calculated by OCLR method from both TCGA-CESC and one GEO dataset. Further investigation discovered a notable relation between mRNAsi and hub genes, as well as their mutation status and infiltration of stroma cells among CESC samples. Moreover, we investigated the underlying functional relevance of mRNAsi-related metabolism genes with the calculated gene modules using WGCNA. Subsequently, we employed shrinkage estimation and univariate analysis to identify the most significant metabolism-related genes for prognosis, forming the 3-stemness-related metabolic gene signature for CESC. Following this, samples from both the TCGA-CESC dataset and GSE44001 external dataset were integrated into the model and stratified based on the risk score to assess the model’s predictive performance and stability in the prognosis prediction. Additionally, we explored the correlations between risk score and relevant clinicopathological characteristics, along with signal pathways.

As a result, we investigated the relationship between cancer stemness and the tumor microenvironment (TME) in CESC and subsequently selected differentially expressed metabolism-related genes (DEMRGs) between high- and low-mRNAsi groups in CESC. Previous literature has highlighted the significant involvement of certain DEMRGs in stemness-related processes. Among 23 DEMRGs (including GAPDH, SLC19A1, TCN1, ARSF, CD320, CERS3, AKR1B10, AKR1B15, SULT2B1, SLC5A1, GNA15, ALOX12B, PLA2G4E, PLOD1, ALDH1B1, HACD1, PLOD2, AP2B1, LPCAT1, GPX8, CSPG4, GCNT1, BCAT1), it was found that six genes (AKR1B10, GNA15, ALDH1B1, PLOD2, LPCAT1, and GPX8) were differentially expressed in both the TCGA-CSEC and GEO datasets. The 23 DEMRGs are involved in Pantothenate and CoA biosynthesis, Lysine degradation, and Arachidonic acid metabolism pathways. AKR1B10 is a NADPH-dependent reductase and is highly expressed in epithelial cells. It serves as a prognostic factor for recurrence following surgical intervention in CESC [16]. ALDH1B1 gene expression exhibited a positive association in CESC [17]. PLOD2 is an effective prognostic marker, which is related to the immune infiltration of cervical cancer [18]. LPCAT1 had never been reported in cervical cancer but was reported to promote gefitinib resistance through upregulation of the EGFR/PI3K/Akt signaling pathway in lung carcinoma [19], and it has been recognized as a promising prognostic biomarker in liver cancer [20]. GPX8 has shown diagnostic potential across various cancers besides cervical cancer, including glioma, kidney cancer, and stomach cancer. Downregulation of GPX8 has been found to suppress the migratory and invasive properties of glioblastoma cells. GPX8 is under the regulation of FOX1 transcription and facilitates the proliferation of gastric cancer cells by activating Wnt signaling pathway [21]. Further investigations into cellular mechanisms regarding the interplay between metabolic systems and CSCs, and interventions targeting pivotal nodes within this interaction, hold promise as a strategy for cancer therapy. Furthermore, we validated four hub genes by IHC in our patients’ samples at protein level.

We further explored the underlying roles of metabolism-related genes in the pathogenesis of CESC. These genes are implicated in key metabolic pathways such as glycolysis, lipid metabolism, and amino acid metabolism. They regulate various aspects of cellular metabolism, such as glucose uptake, lipid synthesis, and amino acid transport, which ultimately impact tumor cell proliferation, invasion, and metastasis. Dysregulation of these metabolic pathways contributes to the aggressive behavior of CESC. Specifically, enhanced glycolysis provides energy for rapid tumor growth, aberrant lipid metabolism supports membrane biogenesis and signaling pathways, and altered amino acid metabolism fuels protein synthesis and cellular proliferation. Understanding the involvement of metabolism-related genes in CESC pathogenesis provides insights into the molecular mechanisms underlying tumor progression and may guide the development of novel therapeutic strategies targeting metabolic vulnerabilities in CESC.

This study revealed that DEMRGs between high- and low-mRNAsi groups in CESC were enriched in several critical signaling pathways. Some of these pathways have been implicated in regulating stemness processes. For instance, Retinol metabolism, Metabolism of xenobiotics by cytochrome P450, and PPAR signaling pathway have all been linked to cancer prognosis and the regulation of CSCs. Specifically, retinoic acid-mediated signaling, which is involved in retinol metabolism and regulated by aldehyde dehydrogenase (ALDH), has been associated with reduced oxidative stress and drug resistance in cancer [22]. The PPAR signaling pathway is related to lymph node metastasis in cervical cancer and is considered to affect the proliferation of other cancers [23]. Additionally, alterations in ascorbate and aldarate metabolism have been observed in leiomyomas of the MED12 and triple wild-type subtypes, suggesting potential implications for tumor development [24]. Specific treatment for abnormal signal pathways driven by protein tyrosine kinase (TK), which is involved in proliferation, metastasis, and growth, has become a promising anti-cancer method for several years [25]. Thiamine metabolism is associated with human papillomavirus (HPV) infection [26]. Thiamine has been linked to cancer due to its influence on various molecular pathways, including matrix metalloproteinases, prostaglandins, cyclooxygenase-2, reactive oxygen species, and nitric oxide synthase [27]. Therefore, a better understanding of CSC metabolic dependence and metabolic communication between CSC and tumor microenvironment is essential for effective cancer treatment. GSEA analysis also confirmed that metabolism-related signaling pathways was involved.

Various cancers have shown interactions between CSCs and surrounding immune cells, leading to alterations in the tumor microenvironment [28]. In this study, we observed that the 8 hub mRNAsi-related genes were significantly correlated with different immune cell populations between high- and low-mRNAsi groups, such as B cells memory, B cells naïve, macrophages M1, mast cells resting, NK cells activated, T cells CD8, and T cells follicular helper in CESC. Especially, CLCA4 was negatively correlated with B cell naïve and plasma cells. These immune cells have been implicated in regulating stemness processes. For instance, CSCs are important in recruiting tumor-associated macrophages (TAMs) and polarizing them towards the M2 phenotype [29]. CSCs induce Treg infiltration, and Treg indirectly regulates the proliferation and expansion of CSCs through angiogenesis and EMT [30]. In our investigation, we observed notable distinctions in the composition of immune cells between high- and low-risk score groups in cervical squamous cell carcinoma and endocervical adenocarcinoma (CESC). Additionally, immune cells exhibited the capacity to identify, target, and eliminate malignant cells, while also potentially influencing the acquisition of stem cell-like properties in a subset of cancer cells. These findings underscored the potential clinical utility of immunotherapeutic strategies leveraging cancer stem cell-cell interactions within the tumor microenvironment.

The combined application of cervical cytology screening, HPV testing, and colposcopy leads to a higher detection rate of precancerous lesions in CESC. Additionally, the widespread adoption of HPV vaccination effectively reduces the risk of CESC. However, the high costs associated with these examinations and the insufficient dissemination of cervical cancer prevention education result in women in developing countries, including China, missing out on widespread screening and vaccination opportunities. Moreover, due to the atypical early symptoms of CESC and limitations in existing diagnostic methods, over 50% of patients present with local infiltration or lymphatic metastasis upon diagnosis, with a 5-year survival rate of less than 17%. In the era of personalized medicine, accurately forecasting the clinical outcome of CESC patients immediately after surgery is crucial for precise treatment. Therefore, there is an urgent need in clinical practice to identify specific biomarkers that can accurately predict the prognosis of CESC patients, enabling the implementation of personalized treatment and follow-up plans to improve patient outcomes. Nowadays, many studies are currently devoted to identifying such biomarkers (including miRNA, lncRNAs, and mRNA signature), with their AUCs at 1-year, 3-year, and 5-year intervals typically around 0.7. In our study, however, the AUCs at 1-year and 5-year intervals are higher, thus enriching and complementing existing research in this field.

Currently, with the rapid advancements in high-throughput whole genome sequencing technology, accessing the vast biological information from large-scale CESC samples has become more feasible, facilitating the exploration of latent information. Although our DEMRGs signature has advantages, like other bioinformatics research, the current work also has some limitations. Firstly, the lack of experimental validation necessitates further investigation into the potential mechanisms of the identified genes. Additionally, our analysis is constrained by the limited availability of TCGA-CESC samples and associated clinical data. Therefore, larger sample sizes are warranted to delineate the functional significance of key prognostic stem cell genes in predicting CESC progression.

## CONCLUSION

In conclusion, our study developed a new stemness-based metabolic miRNAsi signature, establishing it as a promising prognostic marker of CESC. It is worth noting that our metabolic-related gene markers linked the molecular characteristics of CESC stem cells with clinical results, offering insights into potential therapeutic effects and prognostic predictors. In addition, this research method has universal applicability and certain reference value for other cancer types.

## MATERIALS AND METHODS

### Data sources and pre-processing

The methodology of this study involved a systematic approach outlined in Figure 1. The reliable CESC expression profile dataset GSE44001 and TCGA-CESC were sourced from GEO database (https://www.ncbi.nlm.nih.gov/geo/) and TCGA (https://www.cancer.gov/) database by AnnoProbe package (https://github.com/jiangfuqing/GEO-AnnoProbe) and TCGAbiolink package of R software, version 4.0.2, respectively (http://r-project.org/). Both datasets comprised samples from Homo sapiens, with GSE44001 derived from the GPL14951 platform (Illumina HumanHT-12 WG-DASL V4.0 R2 expression beadchip). The GSE44001 dataset consisted of 97 cervical squamous cell carcinoma (CSCC) samples. The information of GEO dataset and TCGA dataset was shown in Table 4. The background signal correction, normalization, and summarization were performed by the affy package. Then the gene expression matrix of the two datasets was obtained. SNP mutation data retrieved from the TCGA database was integrated with corresponding RNA-seq data, and mutation landscape visualization was performed using the maftool package. [4]. Metabolic-related genes were sourced from the Genecards database to perform subsequent analyses (https://www.genecards.org/).

**Table 4:** The information of GEO dataset and TCGA dataset.

**Dataset**	**Data type**	**Platform**	**Sample size**	**Disease**
TCGA-CESC	RNA-seq	Illumina HiSeq sequencing	279	cervical cancer
GSE44001	Expression microarray	Illumina HumanHT-12 WG-DASL V4.0 R2 expression beadchip	300	cervical cancer

### mRNAsi calculation based on gene expression matrix

For all samples in the two datasets, the index mRNAsi of gene stemness was computed based on the dataset matrix by ssGSEA algorithm based on relative expression sequence (REOs) [5], using R-package GSVA [6].

### Screening and functional analysis of DEGs

The mRNAsi of each sample obtained according to the method above was stratified into high-mRNAsi subgroup and low-mRNAsi subgroup based on the level of mRNAsi. The GSE44001 dataset and the TCGA-CESC dataset obtained the DEGs between the two groups through the limma package and Deseq2, respectively [7]. We applied the ggplot2 package to depict the volcanic map of DEGs and used the pheatmap package to depict the heatmap of DEGs to show the DEGs expression. The criteria for selecting DEGs of the GSE44001 dataset are *p* adj < 0.05 and |log2fc| > 1.

### WGCNA

The overall process of WGCNA involves calculating pairwise gene correlations, constructing a hierarchical clustering tree to identify gene modules, determining module significance (MS), and assessing the correlation between gene expression and traits of interest. This includes measuring gene significance (GS) and module membership (MM) to identify relevant gene modules highly correlated with the trait. Finally, modules are selected based on their significance for further analysis.

### Molecular typing construction

Candidate genes were included and subjected to Non-negative Matrix Factorization (NMF) analysis using the R software package NMF. In this study, when a relatively satisfactory consensus map and cophenetic and silhouette coefficients were observed at the same time, the number of runs was set to 40 and the number of clusters was set to 2. The final clustering yielded two distinct molecular subtypes, based on gene expression profiles, which were visualized using a heatmap to display the expression patterns of key genes.

### Prognostic marker screening

The expression profile of each candidate gene was computed according to the TCSC expression data by using the Cox proportional hazards model. Genes related to prognosis were selected by univariate Cox regression analysis, where a hazard ratio (HR) greater than 1 indicated a risk gene, while an HR less than 1 indicated a protective gene. Statistical significance was set at *p* < 0.1. Subsequently, LASSO Cox regression analysis was employed for variable selection and shrinkage within the Cox proportional hazards model. This approach constructs a penalty function to refine the model and improve predictive accuracy. Next, we applied the lasso algorithm to screen possible variables in the Cox regression model to identify important prognostic markers, and we selected a standard error (SE) higher than the minimum standard. A stepwise Cox proportional-hazards (Cox PH) regression model was constructed to search the predictors of overall survival. The gene variables were entered into the model. This stepwise regression approach was employed to optimize the model and enhance its practical utility, with a threshold level set at 5% to identify a concise yet informative model comprising essential genes (markers) and relevant clinical covariates associated with CESC prognosis.

In our study, we used the glmnet package in R for lasso regression and the step function in the survival package for stepwise Cox regression. For lasso regression, common parameter settings include λ chosen via cross-validation (e.g., cv.glmnet function with nfolds parameter) and max iterations set to a sufficiently large value (e.g., 1000). For stepwise Cox regression, entry and exit criteria are typically set at *p* < 0.05, and both forward and backward selection directions can be explored. Finally, by taking the optimized gene expression and relatively estimating the Cox regression coefficient into consideration, we calculated the risk score based on the formula: risk score = (exp-Gene1 × coef-Gene1) + (exp-Gene2 × coef-Gene2) + …… + (exp-Gene × coef-Gene).

Accordingly, samples were stratified into high-risk group and low-risk group according to the given risk score. Kaplan-Meier analysis, supplemented by a log-rank test, was conducted using the survival package to assess overall survival (OS) within the test cohort. Additionally, survival prediction was evaluated through ROC curve analysis. The prognostic or predictive accuracy was quantified by calculating AUC using the pROC package.

### Immune correlation analysis and its correlation with hub gene

Following data upload and filtering (*P* < 0.05), the immune cell infiltration matrix was obtained. Distribution of the 22 immune cell types across samples was visualized using histograms generated with the ggplot2 package. Furthermore, the ggcorrplot package facilitated the creation of heatmaps to illustrate the association between hub genes and immune cell infiltration or immune-related genes. Tumor immune scores, including stromal score, immune score, and estimate score, were computed according to mRNA expression by estimate method from the R package.

### IHC staining

Paraffin-embedded tissue samples were sectioned at 4 μm thickness. Antigen retrieval was conducted by incubating the sections in citrate buffer (pH 6.0) at 100°C in a microwave oven for 15 minutes, followed by natural cooling to room temperature. Following blocking with a mixture of methanol and 0.75% hydrogen peroxide, the sections were sent to incubate overnight with primary antibodies (GNA15, ALDH1B1, LPCAT1, GPX8) at specified dilutions. Subsequently, secondary antibodies conjugated with HRP were applied, followed by washing with PBS and incubation with AEC substrate. Further details of the analysis procedure can be found in our previous publication [14].

### Statistical analysis

Data processing and analysis were performed by R software. For comparisons between groups, statistical significance of continuous variables was assessed using *t*-tests or Mann-Whitney *U*-tests, while categorical variables were compared using chi-square or Fisher’s exact tests. Pearson correlation analysis was used for gene correlations. Survival analyses utilized Kaplan-Meier curves and log-rank tests, with Cox regression for prognostic factors. All reported *P*-values were two-sided, with *P* < 0.05 considered significant.

### Data availability

The data used to support the findings of this research are available from TCGA database (https://cancergenome.nih.gov/); and Gene Expression Omnibus database (https://www.ncbi.nlm.nih.gov/geo/).
